# Administrative Censoring in Ecological Analyses of Autism and a Bayesian Solution

**DOI:** 10.1155/2011/202783

**Published:** 2011-05-08

**Authors:** Scott M. Bartell, Thomas A. Lewandowski

**Affiliations:** ^1^Program in Public Health and Department of Statistics, University of California, Irvine, 2241 Bren Hall, Irvine, CA 92697-1250, USA; ^2^Gradient, Seattle, WA 98101-1248, USA; ^3^Department of Health and Nutrition Sciences, Brooklyn College, Brooklyn, NY 11210, USA

## Abstract

Widely cited ecological analyses of autism have reported associations with mercury emissions, with precipitation, and race at the level of counties or school districts. However, state educational agencies often suppress any low numerical autism counts before releasing data—a phenomenon known as “administrative censoring.” Previous analyses did not describe appropriate methods for censored data analysis; common substitution or exclusion methods are known to introduce bias and produce artificially narrow confidence intervals. We apply a Bayesian censored random effects Poisson model to reanalyze associations between 2001 Toxic Release Inventory reported mercury emissions and 2000-2001 autism counts in Texas. Relative risk estimates for autism decreased from 4.44 (95% CI: 4.16, 4.74) per thousand lbs. of air mercury emissions using a naive zero-substitution approach to 1.42 (95% CI: 1.09, 1.78) using the Bayesian approach. Inadequate attention to censoring poses a serious threat to the validity of ecological analyses of autism and other health outcomes.

## 1. Introduction

The prevalence of autism has been increasing in the US over the last few decades, but its causes are not well understood [1]. Several ecological analyses of autism have appeared in the last five years, reporting correlations with reported mercury emissions [2, 3], with precipitation [4], and with race [5]. These study findings have been cited extensively by other peer-reviewed publications [6] and widely circulated by the popular press [7, 8]. Although it is well known that ecological studies may be affected by aggregation bias when used to estimate individual level effects [9], ecological studies of medical conditions such as autism are typically susceptible to an additional hazard that is seldom recognized: censoring of low disease counts.

Autism is not a reportable disease, but states do track and report the total number of students receiving special education services for conditions such as autism to the US Department of Education under the 1990 Individuals with Disabilities Education Act (P.L. 101–476, Section  618). Statewide totals are routinely published by the US Department of Education [10], but prevalence by school district or county must be obtained from each state's department of education. Countywide environmental releases of mercury are also available publicly through the US Environmental Protection Agency Toxic Release Inventory, through a mandatory reporting system that includes any facility emitting 10 lbs. or more of mercury per year. 

Educators have legal and moral obligations to protect the privacy of students and their educational records. Some states impose limits upon enumeration of small numbers of students receiving particular special education services in order to ensure privacy for these students. For example, the Texas Educational Agency provided us with exact autism counts for districts with 5 or more students with autism and for districts with no students with autism, but it used a special code (−999) for districts with 1–4 students with autism. 35% of the districts fell into the latter category requiring the special code (Table 1). Similarly, Waldman et al. [4] reported that Oregon censored the autism counts (i.e., substituted a special code for the actual counts) for counties with fewer than 10 students with autism at any one year of age.

These autism counts take the form of “administratively censored” outcomes. Likelihood-based statistical methods for administratively censored outcomes have long been available for classical parametric fixed effects models [11] and are widely implemented for well-known distributions in major statistical packages. However, previous ecological studies of autism have cited statistical methods such as mixed effects Poisson models and ordinary least-squares regression that are not designed to handle censored observations [2–5]. The absence of any explicit description of methods for handling censored observations in these previous publications suggests that censored values were deleted or substituted with fixed values such as zeros. Indeed, one might substitute zeros after incorrectly assuming that a missing autism count indicates that a school district had no children with autism to report. 

Palmer et al. [2, 3] fit random effects Poisson models associating 2000-2001 school district autism counts and 2001 countywide total mercury releases, adjusting for percent white, district wealth, percent economically disadvantaged, and urbanicity, but they did not discuss censoring. We were able to compile these variables for 1029 of the school districts, and previously reanalyzed the data for these districts in various years using the model described by Palmer et al. [2]. We previously reported similar results to Palmer's for 2000-2001 when using the zero substitution approach, with RRs of 1.29 to 2.03 per 1000 lbs of total mercury releases [12], depending on the estimation algorithm, versus Palmer's originally reported RR of 1.61. However, when we substituted larger fixed values (threes) for the censored counts, our RR estimates decreased to values near unity [12]. Statistically significant associations between mercury emissions and autism did not persist in later years. 

Substantial differences between the results of *ad hoc* fixed value substitution methods indicate that the method of handling censored counts is critical in this setting, motivating the following analysis.

## 2. Methods

In general, the contribution of each interval censored observation to the likelihood is Pr (*L* < *Y*_*i*_ ≤ *U*), where *Y*_*i*_ is a random variable describing the count in district *i* and (*L*, *U*] is the interval in which the censored value lies. The following statements comprise a censored Poisson random effects model for the ecological association between mercury releases and autism using the Texas data:

(1) $$\begin{array}{c} \text{ Pr}\left(Y_{i}\;=\;y_{i}\right)\;=\frac{e^{-\lambda_{i}}\lambda_{i}^{y_{i}}}{y_{i}!}\;\text{for}\;y_{i}=0\;\text{or}\;y_{i}>4\\ \begin{array}{cc} & \text{Pr}\left(0<Y_{i}\leq 4\right)\\ & \;\;=\overset{4}{\underset{y_{i}=1}{\sum }}\frac{e^{-\lambda_{i}}{\lambda_{i}}^{y_{i}}}{y_{i}!},\\ & \;\;\text{for}\;\text{censored}\;\text{counts}\;\left(y_{i}\;\text{coded}\;\text{as}\;-999\right), \end{array}\\ \begin{array}{cc} \ln\;\left(\lambda_{i}\right) & =\ln\;\left(\text{DistrictSiz}{\text{e}}_{i}\right)+\mu +\beta_{0j}+\beta_{1}\text{Mercur}{\text{y}}_{i}\\ & \;+\beta_{2}\text{Whit}{\text{e}}_{i}+\beta_{3}\text{Taxbas}{\text{e}}_{i}+\beta_{4}\text{Eco}{\text{n}}_{i}+\beta_{5}\text{Urba}{\text{n}}_{i}\\ & \;+\beta_{6}\text{Suburba}{\text{n}}_{i}+\beta_{7}\text{Other}\;\text{Urbanicit}{\text{y}}_{i}, \end{array}\\ \beta_{0j}\sim N\left(0,\sigma_{0}^{2}\right), \end{array}$$

where *y*_*i*_ is the observed autism count for school district *i*, *λ*_*i*_ is the Poisson rate parameter modeled as an exponentiated linear combination of seven covariates and an offset for the log of the number of students in the district, and *β*_0*j*_ is a random effect of county *j* (many counties have more than one school district). *μ*, *σ*_0_^2^, and the *β*s are unknown parameters, and *β*_1_ is the parameter of interest: the log RR for autism associated with each 1000 lbs increase in total mercury emissions. *N*(0, *σ*_0_^2^) denotes a normal distribution with mean 0 and variance *σ*_0_^2^. 

Although censored Poisson fixed effects regression is widely implemented by major statistical software packages, interval-censored Poisson random effects regression is a newer area of statistical research. Moreover, frequentist Poisson random effects regression algorithms rely on differing likelihood approximations that are known to produce disparate estimates for these particular data [12] and for rare outcomes in general [13]. We use a Bayesian approach for parameter estimation instead, requiring the specification of prior distributions for the unknown parameters. We chose relatively uninformative priors

(2) $$\begin{array}{c} \mu \sim N\left(-\;6,\;10\right),\\ \beta_{k}\sim N\left(0,100\right)\;\text{for}\;\text{all}\;k,\\ \sigma_{0}^{2}\sim \text{Uniform}\;\left(0,\;50\right). \end{array}$$

We implemented the model using WinBUGS 1.4.3, a generalized environment for conducting Bayesian analyses using Markov chain Monte Carlo methods, as well as the R2WinBUGS library in R. WinBUGS code is provided (see Algorithm 1). At least, 100,000 iterations were used in each analysis for each of 3 chains; 50% of the iterations were discarded for burn-in. Convergence of the sampling chains was assessed using plots and the R-hat statistic, which was less than 1.01 for each posterior RR for mercury and less than 1.05 for all other parameters. 

For comparison purposes, naive approaches relying on either exclusion of censored observations or fixed-value substitution for censored observations were conducted using mixed effects Poisson regression in R as described previously [12] but with a more recent version of the lmer library (version 0.999375-31). 

We also repeated all analyses for air mercury emissions in place of total mercury emissions. Air emissions are arguably a better proxy for concurrent exposure than total mercury emissions for reasons described in our previous analysis [12] and later in this paper.

## 3. Results

The resulting Bayesian posterior mean RR estimate per 1000 lbs of reported total mercury releases using the 2000-2001 data is 1.18 (95% confidence interval (CI): 1.07, 1.32). In contrast, the naïve zero-substitution method yields an RR estimate of 2.02 (95% CI: 1.96, 2.09) per 1000 lbs. of total mercury releases.  Table 2 shows the complete set of parameter estimates for the Bayesian censored likelihood analysis and for the naïve substitution approach using various fixed values and for the exclusion approach. Although some of the naïve approaches produce reasonable central estimates, they produce artificially narrow confidence intervals.

For air mercury emissions, the posterior mean RR estimate is 1.42 (95% CI: 1.09, 1.78) per 1000 lbs. of mercury. In contrast, the zero-substitution approach yields an RR estimate of 4.44 (95% CI: 4.16, 4.74) per 1000 lbs. of mercury. Complete results for air mercury emissions are shown in Table 3. 

We checked for sensitivity to the choice of the prior by repeating the air mercury analysis with the prior for *β*_1_ centered on log(4.44). This analysis yielded nearly identical results (RR = 1.42; 95% CI: 1.10, 1.80) to the primary analysis, suggesting that our choice of prior distribution for the effect of mercury is not overly influential.

## 4. Discussion

Our results using the full censored likelihood indicate that the ecological association between total mercury emissions and 2000-2001 autism in Texas school districts may be much smaller than previously reported. Published associations between precipitation and autism are also dependent on censored data and could be affected by similar biases. RR estimates for the ecological association using the censored likelihood were fairly similar to the results using the *ad hoc* substitution approach when values near the middle of the censoring interval were chosen. However, confidence intervals were (appropriately) wider for the Bayesian likelihood-based approach than for the substitution approaches. Exclusion of all censored values produced similar central RR estimates to the Bayesian approach but with artificially narrow confidence intervals.

However, all results should also be viewed as ecologic effect estimates that may not be representative of individual level effects due to aggregation bias. Another consequence of the group-level analysis is that uncertainty regarding individual-level associations between mercury exposure and autism in Texas is much greater than suggested by the group-level confidence intervals reported here and in previous publications [3, 5, 12].

**Figure 1:**
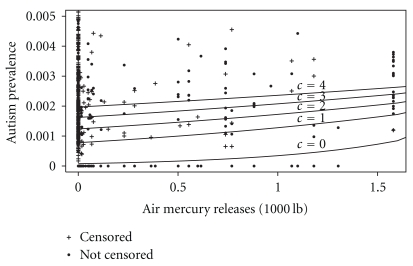
Autism prevalence versus Toxic Release Inventory reported air mercury emissions, with threes substituted for censored values (*c* = 3) for all data points. Lines show the covariate-adjusted random effects Poisson model predictions using five different fixed value substitution approaches (substituting different values of *c* for the censored counts).

These results were surprising to us given that the cutoff for censoring was so low (5 students), but they may be explained by an apparent association between mercury emissions and the presence of censoring, whereby those districts with small autism counts tended to have lower mercury emissions. This effect may be better understood through inspection of Figure 1, which shows the data and model fit using a variety of fixed value substitution approaches. Because many of the censored values occur in counties with low mercury emissions, the overall intercept from the mixed effects Poisson regression is highly sensitive to the choice of fixed value substituted for the censored observations. In contrast, there are relatively few censored observations in counties with higher mercury releases, leading to a more stable position of the right-hand side of the prediction curve. The result appears to be a forcing of the slope (the log relative risk) to higher values when the intercept is lower.

Although both total mercury releases and air mercury emissions are poor surrogates for individual mercury exposures, air mercury emissions may be a better proxy for concurrent exposures than total mercury releases. Total mercury releases include landfill disposals and other releases that may not lead to widespread or immediate public exposure, especially if compliant with the 1976 Resource Conservation Recovery Act (P.L. 94–580) and its 1984 amendment. In contrast, air mercury emissions are more widely and rapidly dispersed with wind and rainfall and are, therefore, of the two, more plausible sources of human exposure in the short term. Surface water mercury emissions may also be quickly dispersed, but these emissions were relatively small in Texas in 2001: only 25% of Texas counties reported any surface water mercury emissions, and the largest annual release was 16 lbs. Air mercury releases occurred in much larger quantities, with reported releases in most Texas counties, a mean annual release of 288 lbs., and a maximum annual release of 1579 lbs. Although these analyses are limited by the ecological nature of the data and the crude measure of exposure, we believe that the air mercury emissions are the more relevant measure within these limitations. Researchers considering similar analyses should also consider time-lagged comparisons of autism counts with emissions/exposure estimates from previous years to allow enough time for environmental transport, autism development, and diagnosis to have occurred [12]. 

The three substitution and exclusion approaches happen to provide a rough approximation to the central RR estimate for these particular data, but likelihood-based approaches for handling censored data produce more realistic confidence intervals and have a stronger theoretical basis. All of the previous and current approaches, however, depend on common model assumptions such as Poisson distributed counts, log linearity of the predictors, and adequate control of confounding. The third assumption is of most concern in the present analysis, given the limited understanding of autism risk factors and a lack of any individual-level data on mercury exposure or confounders. 

Ecological studies of autism are not the only studies where censored disease counts are a threat to validity. All special education category counts reported by Texas are administratively censored, and the same practice appears to be followed by some other states. Other count data reported by states may be affected by similar issues. Moreover, efforts to protect privacy under the Health Insurance Portability and Accountability Act of 1996 (P.L.104–191) may lead to administrative censoring of medical surveillance data when counts are extremely low. With the widespread computerization of large data sets, ecological analyses are becoming ever easier to conduct and will likely appear more frequently in the scientific literature despite their limitations. 

Censoring also arises in chemical concentration measurement, another common issue in environmental health, through reporting of low concentrations as below the “limit of detection” (LOD). If censored values are excluded, calculated means will generally be biased upwards in this setting. Environmental health researchers have a long tradition of substituting zeros, LOD/2, LOD/$\sqrt{2}$, or LOD for these censored values, treating the substituted values as if they were actually observed. The substitution approach is easy to implement but is inferior to formal likelihood-based censored data analysis in that it may also produce biased estimates and always fails to capture the uncertainty associated with measurements below the LOD, such as in calculating confidence intervals [14]. However, the adverse effects of substitution or exclusion may be negligible when both of the following conditions are met: (1) few samples are below the cutoff for censoring and (2) the cutoff for censoring is small relative to most of the measurements.

## 5. Conclusions

Our analyses indicate that previously reported ecological associations between mercury and autism in Texas are likely to have been overestimated due to inadequate statistical analysis of censored autism counts. Researchers should be aware of the issue of administrative censoring of disease counts and how ad hoc substitution methods can introduce bias and underestimate uncertainty in effect estimates. Bayesian methods offer a potential solution to the problem, do not rely on likelihood approximations, and are not difficult to implement.

## Figures and Tables

**Algorithm 1:**
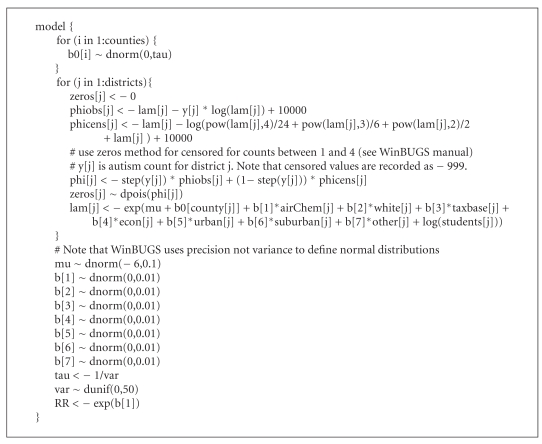
WinBUGS code.

**Table 1:** Distribution of 2000-2001 autism counts* for Texas school districts.

Students with autism	Number of school districts
0	451
1–4 (censored)	362
5	18
6	16
7	16
8	15
9	7
10	10
11–20	53
21–50	44
>50	37

*For brevity, this table summarizes counts greater than 10 using several categories though exact counts are available for districts with more than 4 students with autism. Only counts of 1 to 4 are censored.

**Table 2:** Effect estimates per 1000 lbs of Toxic Release Inventory reported total mercury releases using various censored data methods.

Approach	RR	95% CI
Bayesian censored likelihood	1.18	1.07, 1.32
Zero substitution	2.02	1.96, 2.09
One substitution	1.26	1.17, 1.36
Two substitution	1.18	1.08, 1.28
Three substitution	1.14	1.02, 1.27
Four substitution	1.11	0.96, 1.29
Exclusion	1.16	1.14, 1.18

**Table 3:** Effect estimates per 1000 lbs of Toxic Release Inventory reported air mercury emissions using various censored data methods.

Approach	RR	95% CI
Bayesian censored likelihood	1.42	1.09, 1.78
Zero substitution	4.44	4.16, 4.74
One substitution	1.64	1.40, 1.93
Two substitution	1.39	1.15, 1.69
Three substitution	1.28	1.00, 1.63
Four substitution	1.2	0.87, 1.65
Exclusion	1.37	1.31, 1.42

## Abbreviations

CI: Confidence interval
LOD: Limit of detection
RR: Relative risk.
